# Long-Read Sequencing of Chicken Transcripts and Identification of New Transcript Isoforms

**DOI:** 10.1371/journal.pone.0094650

**Published:** 2014-04-15

**Authors:** Sean Thomas, Jason G. Underwood, Elizabeth Tseng, Alisha K. Holloway, on behalf of the Bench To Basinet CvDC Informatics Subcommittee

**Affiliations:** 1 The Gladstone Institutes, San Francisco, California, United States of America; 2 Department of Epidemiology and Biostatistics, University of California San Francisco, San Francisco, California, United States of America; 3 University of Washington, Seattle, Washington, United States of America; 4 Pacific Biosciences, Menlo Park, California, United States of America; Heart Science Centre, Imperial College London, United Kingdom

## Abstract

The chicken has long served as an important model organism in many fields, and continues to aid our understanding of animal development. Functional genomics studies aimed at probing the mechanisms that regulate development require high-quality genomes and transcript annotations. The quality of these resources has improved dramatically over the last several years, but many isoforms and genes have yet to be identified. We hope to contribute to the process of improving these resources with the data presented here: a set of long cDNA sequencing reads, and a curated set of new genes and transcript isoforms not currently represented in the most up-to-date genome annotation currently available to the community of researchers who rely on the chicken genome.

## Introduction

Research into the development, structure and function of different aspects of metazoan biology has been greatly assisted by the genomics tools that have become available over the last several years. In addition to the sequence of bases that constitute a genome, the locations and boundaries of genes and their exons must also be identified. Furthermore, many genes exhibit alternate splicing such that a few exons may exhibit a large degree of combinatorial possibilities. These transcript isoforms must also be detected in order to have a thorough set of genome resources. This annotation process typically requires a combination of computational and experimental approaches. All of the important work being done now to uncover the regulatory mechanisms that control when and how genes are active rely heavily on a foundation built with solid genome assemblies and annotations. Humans and several key model organisms have quite good genome assemblies [1]–[5] with other associated resources including high quality annotations for transcript isoforms and even the locations of functional regulatory elements in various cell and tissue types [6]–[9].

Resources for other model organisms are also being developed. The UCSC genome browser [10] currently contains assemblies and annotations for dozens of eukaryotic organisms. These resources are in various states of accuracy and completeness. In the case of the genome for *Gallus gallus*, the common chicken, the genome was initially published in 2004 [11] and has been periodically updated since, with the latest being the ‘galGal4’ assembly published in late 2011. The most up-to-date transcript annotations for these assemblies come from RefSeq [12] and Ensembl [13], [14]. The RefSeq annotations are well-founded but only contain a fraction of the total number of genes and isoforms. The Ensembl annotation is much more thorough but is not yet complete as it does not contain annotations for regions seen to be transcribed in previously-published data [15]. The current annotations also contain fewer genes and isoforms than those of the more complete human and mouse genomes [1], [2], [16], though it is possible that there are systematic differences in the number of alternate isoforms seen in avian and mammalian genomes.

The chicken has been used as a model for embryogenesis for thousands of years [17], and notable scientists such as Aristotle, Harvey, Pasteur and Darwin used chickens as models for embryology, circulation, infection and evolution. The chicken continues to be an extremely useful model organism in many areas including aiding our understand of the molecular mechanisms behind heart development [18].

For these reasons a collaboration was created between the Cardiovascular Development Consortium of the Bench to Bassinet Program and Pacific Biosciences to help improve the chicken genome annotation. By generating new long-read sequences and incorporating existing short-read and EST sequences, we identified thousands of transcript isoforms as well as hundreds of genes not currently included in the Ensembl annotations. It is our expectation that the results presented here will serve as a resource for the community of researchers who rely on the chicken genome, and that our methodology will help others whose goal it is to improve the annotations of other model organism genomes.

## Materials and Methods

### Illumina data

The following Gallus gallus RNAseq datasets made from the respective brains, cerebellums, hearts, livers, kidneys and testes of adult chickens, were retrieved from the NCBI Sequence Read Archive [15]: SRX081869, SRX081870, SRX081871, SRX081872, SRX081873, SRX081874, SRX081875, SRX081876, SRX081877, SRX081878, SRX081879, SRX081880. TopHat2 [19] (v2.0.9, default settings) was used to align these 75 bp sequences to the galGal4 genome assembly and to identify exon junctions. Trinity [20] (2011-11-26 release, default settings) was used to assemble short-read sequences into transcripts.

### Collecting embryonic chicken heart RNA

Chicken eggs representing a cross between a White Leghorn rooster and a Rhode Island Red hen were stored at 4°C after laying and then incubated in Genesis Hova-Bators until tissue was harvested. Hearts were dissected from embryos at HH stages 18–20, 25, and 32 and immediately flash frozen in N2. The hearts were homogenized and suspended in TriZol (Life Technologies) by repipetting, and then total RNA was extracted into the aqueous phase, precipitated with isopropanol, washed with 75% ethanol and then resuspended into nuclease free water.

### RNA purification and cDNA synthesis

mRNA were purified using the Strategene Absolutely mRNA purification kit: Briefly, the RNA were hybridized to oligo-dT magnetic beads, separated from solution on a magnetic stand, washed, and then resuspended into the kit's elution buffer.

First strand cDNA synthesis was performed using the SMART cDNA kit (Clontech): The first cDNA strand was synthesized from purified poly-A RNA using the SMARTScribe MMLV Reverse Transcriptase (Clontech), the CDS III oligo-dT primer and the SMART IV primer for template switching in order to add a consistent 5′ site for LD-PCR amplification using the CDS III primer and the 5′ PCR Primer. CDSIII primer: 5′-ATTCTAGAGGCCGAGGCGGCCGACATG-d(T)30N–1N-3′ (N = A, G, C, or T; N–1 = A, G, or C)

SMART IV oligonucleotide: 5′-AAGCAGTGGTATCAACGCAGAGTGGCCATTACGGCCGGG-3′

5′ PCR Primer: 5′-AAGCAGTGGTATCAACGCAGAGT-3′

### Library preparation and sequencing

The cDNA was run on an agarose gel and four separate size ranges were fractionated: 0–1 kb, 1–2 kb, 2–3 kb, and over 3 kb. Each size fraction was extracted from the gel and SMRTbell libraries were created using the DNA Template Library Preparation kit (Pacific Biosciences): The cDNA was cleaned using Ampure beads and the ends were repaired. Blunt hairpin adapters were then ligated to the insert cDNA, exonucleases were added to remove failed ligation products, and SMRTbell templates with cDNA inserts were purified. The sequencing primer and then the polymerase were then sequentially annealed to the SMRTbell templates using the DNA Polymerase Binding kit (Pacific Biosciences). The MagBead loading kit was used to load annealed templates onto a Pacific Biosciences RS II sequencer, and sequencing was performed for each template library using the DNA Sequencing kit (Pacific Biosciences). Sequences containing both 5′ and 3′ adapters were identified, and the adapters and poly-A/T sequences were trimmed. The resulting sub-reads were then mapped using GMAP [21] (2012-07-20 release, default settings) to the galGal4 genome assembly [11]. Files containing the full length reads used in this study are available through the sequence read archive (SRA accession: SRP038897).

### Transcript isoform validation

To identify sequences most likely to represent actual transcripts that are not currently represented in the most recent Ensembl 74 galgal4 annotation, the long-read sequences were subjected to validation steps after mapping. First, each exon junction was checked for proper splice acceptor (AG|N) and splice donor sites (N|GT). Second, each junction was checked against the database of exon junctions derived from Illumina short-read sequencing (described above). Multi-exon sequences that contained only validated junctions were retained. A junction code was created for each transcript isoform representing the string of junctions that it contained. The isoform codes were merged together to remove duplicate isoform fragments, and then the furthest transcription start and stop sites were assigned to each isoform code in order to handle the observed variability in exact transcription start and stop sites between isoforms with the exact same exons. Those isoforms with codes that were not present among the galGal4 Ensembl annotations were retained. The codes representing new isoforms were converted back into chromosomal locations, and those isoforms were intersected with Ensembl genic regions to identify those transcript isoforms that represented new gene regions.

## Results, Discussion and Conclusions

### Broad coverage of embryonic heart transcripts by new long-read sequencing data

**Figure 1:**
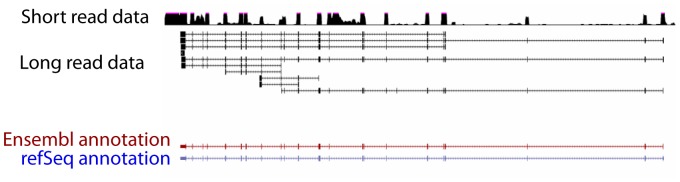
Long-read sequences map accurately to the chicken genome. Shown in this UCSC genome browser image is one example of a long-read alignment shown alongside the corresponding short-read data for this region as well as existing RefSeq and Ensembl annotations.

After extracting RNA from embryonic chicken hearts and creating cDNA libraries, the libraries were sequenced yielding 1,849,786 cDNA sequencing reads. Of these, 1,566,465 (85%) mapped to the galGal4 genome, showing accurate mapping of these reads (e.g. Figure 1). The average mapped transcript length was 1,076 bp, compared to an average of ∼2,400 bp for all Ensembl annotations, when corrected for estimated transcript abundance. This reflects the reality that even with long-read sequences, we were unable to fully sequence the entire lengths of long transcripts. Truncation of transcripts could occur from RNA degradation, mechanical shearing of the sample, incomplete PCR amplification, or loading bias of the sequencer.

**Figure 2:**
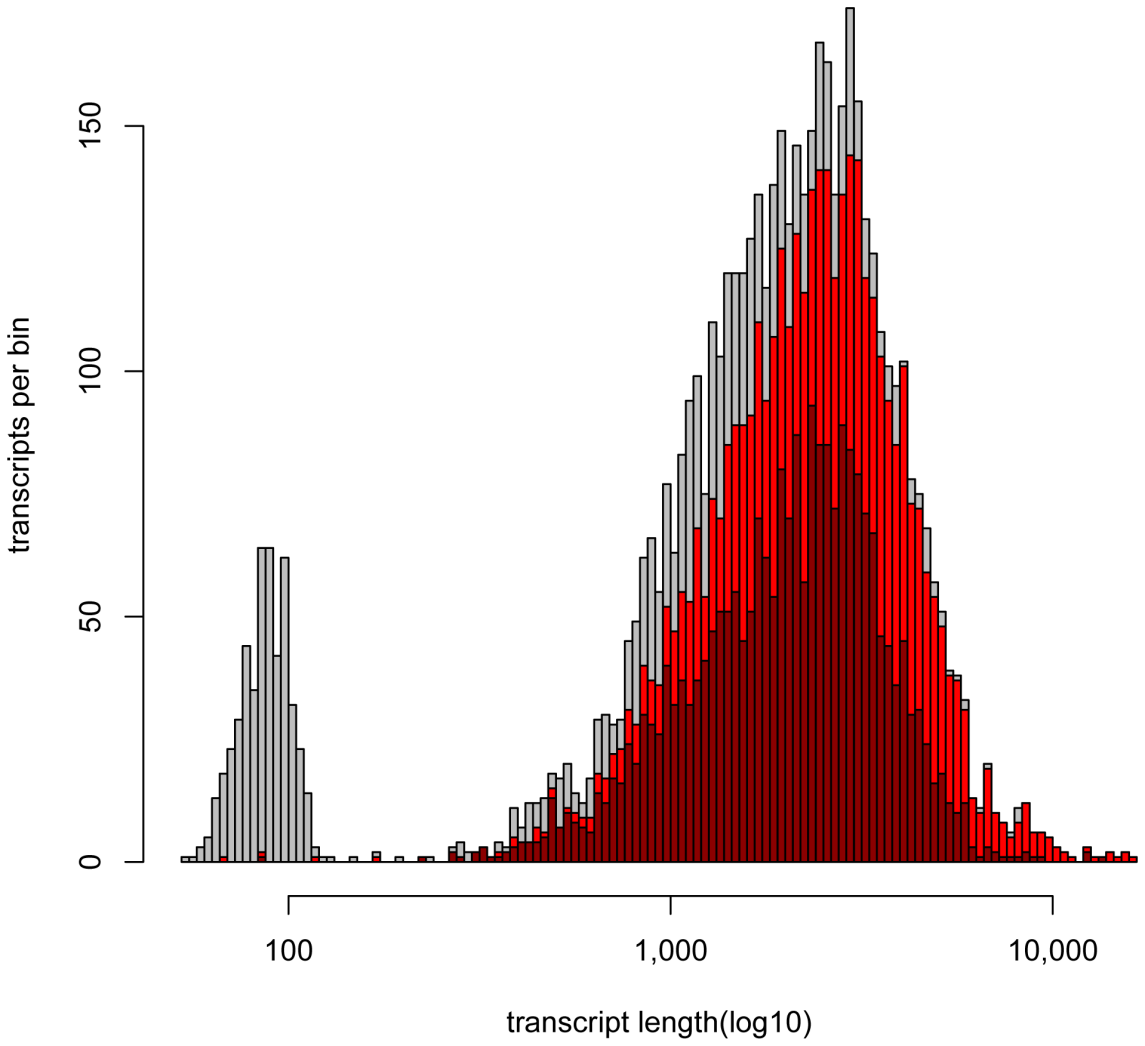
Broad coverage of existing chicken genes using long-read sequencing. Along the x-axis representing transcript length is a histogram of the number of RefSeq transcripts within a given range of lengths (grey). A similar histogram is shown for those transcripts that overlap each RefSeq annotation by any amount (red), or by more than 90% of the gene length (dark red).

To estimate what percentage of the transcriptome these sequences represented, we compared the coverage of these sequences to the per-base coverage of the entire RefSeq annotation (Figure 2). RefSeq was used for this analysis because its annotations represented high-confidence genes conserved across numerous animal species. Of all RefSeq genes, 81% had some overlap with the new sequencing data (Table 1). That number is on par with the fraction of genes that are expected to be expressed in heart tissues, based on the relative coverage of heart RNAseq datasets compared to all RNAseq datasets from chicken (data not shown). This observation suggests that the long-read sequences represent at least partial coverage of almost all chicken genes that are expressed in embryonic chicken hearts. However, only 42% the annotation set was covered to at least 90% of the read length. An additional sequencing run that added over 100 k sequences did little to improve the coverage, suggesting that we were approaching the saturation point of the libraries' complexities. Future efforts to improve the chicken genome would provide greater coverage and across a broader range of tissue types and developmental stages.

**Table 1:** Bulk gene level analysis.

	total #	RefSeq	Ensembl	PacBio	Trinity	EST
RefSeq	5117	x	99%	81%	82%	55%
Ensembl	14282	46%	x	75%	76%	53%
PacBio	40327	29%	55%	x	52%	25%
Trinity	32331	32%	61%	64%	x	37%
EST	11906	38%	76%	75%	84%	x

Each cell along a row represents the percentage of elements (whose number is listed in the first column) that is overlapped (by any amount) of an element from the set of data represented in the column header. For example, 99% of RefSeq annotations were overlapped by Ensembl annotations.

### Overall agreement between long and short-read sequencing

Short-read chicken RNAseq datasets were aligned to the galGal4 genome assembly, resulting in 206,564,870 tags, which were then assembled into contiguous transcripts as described in ‘Materials and Methods’. The overlaps between the long and short-read sequences along with existing isoform annotations from Ensembl and RefSeq are presented in Table 1. More than 75% of the Ensembl annotations were covered by either the long or short-read datasets, while the RefSeq and Manchester EST data [22] covered 46% and 53%, respectively. The similarities in the percentages with which the long and short-read datasets overlap existing annotations suggests good agreement, though they remain incomplete due to lack of RNA sampling and saturation across the many different tissue types. When the long and short-read sequences were compared to each other, there was still quite good agreement, with the long-read sequencing covering more than 60% of the annotations based on short-read data. The short-read data overlapped 52% of the long-read data. Interestingly, the Ensembl annotations covered 99% of the RefSeq annotations, while both sequencing data annotations captured just over 80%, which is possibly due to the relatively limited set of tissues from which the samples were taken.

**Figure 3:**
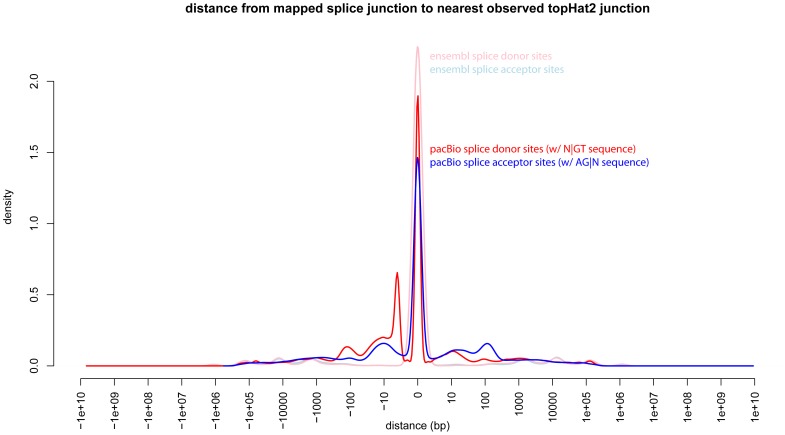
Validation of mapped long-read splice sites. TopHat2 was used to identify observed splice junctions from the short-read data. The light red and light blue lines show the distribution of distances from Ensembl-annotated splice sites to the experimentally observed splice sites. Both of these lines peak heavily at 0 indicating the degree of agreement between these orthogonal datasets. Splice sites annotated from long-read sequencing (blue and red), also show overall agreement, with a small peak of misidentified splice donor sites within 10 bp of the accurate one, which is possibly due to alignment errors near sites with multiple possible splice donor sites.

The above observations suggest quite good agreement on the level of gene regions. To explore how well the splice donor and acceptor sites agree between the short and long-read datasets and the Ensembl annotations, we identified all splice donor sites present in 1.) junctions identified among the short-read sequences, 2.) junctions identified from the Ensembl annotations and 3.) junctions identified from the long-read sequences. Junctions identified from the short-read data match the locations of annotated splice acceptor and donor sites (Figure 3), and for the most part, the long-read data agrees as well. However, approximately 10% of the long sequence reads mapped beyond the ‘correct’ donor site to a downstream ‘GT’ sequence. The origin of this error seemed to be in the process by which the GMAP aligner identifies exon boundaries for sequences with a higher rate of indels. The use of short-read data to validate the splice sites eliminates any effects this mapping error might have on exon boundary identification in the long-read sequences.

### Identification and characterization of new transcript isoforms and gene regions

**Figure 4:**
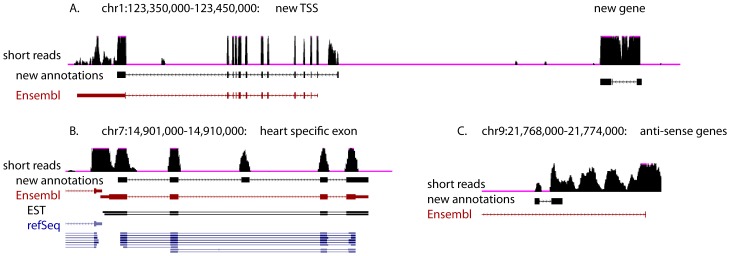
Identification of new isoforms and genes. Shown in this image from the UCSC genome browser are examples of the new genes and isoforms identified from among the short and long-read annotations: A. This region carries two distinct annotations, one for an alternate transcription start site (TSS), and another for a completely new gene that is currently unannotated. B. A heart-specific isoform of the FKBP7 gene. C. Both long-read and short-read data support the existence of transcripts going in opposite directions in this region of chromosome 9.

The short-read and long-read datasets were integrated and used to generate a list of validated transcript isoforms (see Materials and Methods), which were then compared to the Ensembl annotations in order to identify new transcript isoforms (e.g. Figure 4). In all, 9,221 new annotations were generated (Table 2), corresponding to 5,930 different genic regions and included 5,299 currently-unannotated exons. 539 of these regions represent genes without current Ensembl annotations. Searches against the entire RefSeq database only revealed human homologs of three of these genes: FOXE3 (chr6:32,765,038-32,766,317), RASA1 (chrW:242,630-345,306) and FAM179b (chr5:58,707,815-58,739,103). FOXE3 is an important developmental regulator that, in humans, is involved with correct ocular lens formation [23]. RASA1 is a Ras inhibitor linked in humans to angiogenesis through its interactions with the miR-132 microRNA [24]. In chickens there appears to be two paralogs, one of which is annotated on chrZ, while the other is on chromosome W immediately upstream of SMAD2. The lack of overlap with other annotation sets indicates that many genes currently lack any annotations among all of the known datasets.

**Table 2:** New Isoforms and genes.

Ensembl genes/transcripts	14,282/16,743
RefSeq genes/transcripts	6,193/5,117
all new annotations	**9,221**
genes with new annotations	5,930
new genes (not in Ensembl)	539
isoforms for new genes	656
exons in new genes	2,337
new exons in known genes	5,299

**Figure 5:**
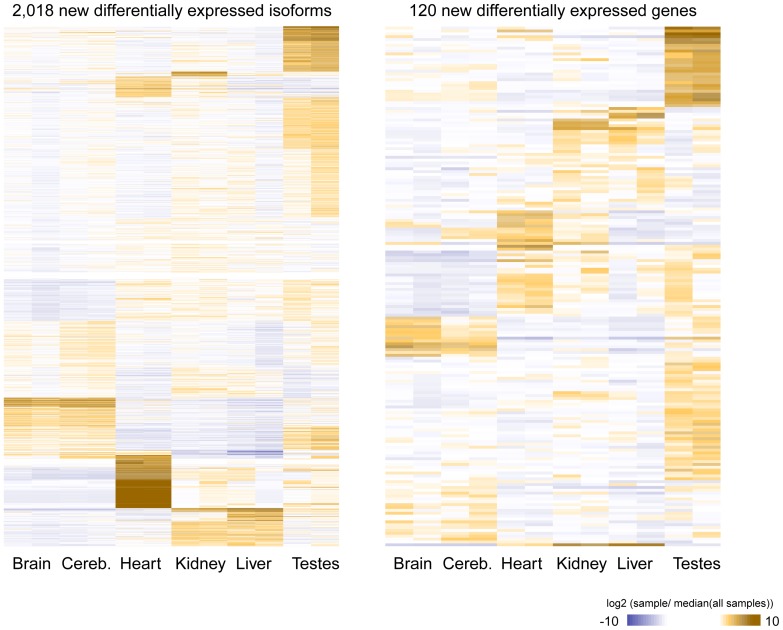
New isoforms and genes with tissue-specific expression. The relative expression of (a) the 2,018 differentially expressed isoforms and (b) the 120 new differentially expressed gene annotations are provided across chicken adult brain, cerebellum, heart, kidney, liver and testes datasets, along the scale provided.

To determine in which tissues the new isoforms might be most enriched, differential RNA analysis was performed using the short-read sequences. 2,018 of the new isoforms were differentially expressed between one or more of the following tissues: brain, cerebellum, heart kidney, liver and testes. A cluster of these differentially expressed isoforms showed enriched expression in hearts (Figure 5a), as was expected given that 20% of the short-read sequences came from adult heart tissue and that all of the long-read sequences came from embryonic heart tissue. In addition, there were three other major clusters of tissue-specific expression. One cluster was enriched in the brain and cerebellum, while the second was enriched in kidneys and liver. The last set of isoforms was enriched in testes. Almost identical clusters were observed among the set of 181 completely new gene regions that were differentially expressed (Figure 5b). Given that the samples used were from a limited number of tissue types, it is likely that more extensive exploration of transcript products will yield many more genes and isoforms specific to those tissues.

### Summary

Using long-read sequences generated from embryonic chicken hearts in combination with short-read sequences from 5 different adult chicken tissues, we were able to contribute over 9,000 new transcript isoforms to the most complete chicken genome annotation, including the identification of more than 500 genic regions without current annotation in Ensembl. While searches against other databases yielded homologs for three of the new gene regions, FOXE3, RASA1, and FAM179B the remaining genes remain uncharacterized, including the 121 gene regions that exhibited tissue-specific expression and might play key roles in chicken biology.

## Supporting Information

File S1 **A bed formatted file containing the galGal4 locations of isoforms for gene regions not found in the current Ensembl annotation.**
(BED)

File S2 **A bed formatted file containing the galGal4 locations of all isoforms not found in the current Ensembl annotation.**
(BED)

File S3 **A bed formatted file containing the galGal4 locations of isoforms within new genic regions not seen in the current Ensembl annotation that exhibited differentially expression among the different tissues examined in this study.**
(BED)

File S4 **A bed formatted file containing the galGal4 locations of the new isoforms not seen in the current Ensembl annotation that exhibited differentially expression among the different tissues examined in this study.**
(BED)
